# Understanding the role of emotion in decision making process: using machine learning to analyze physiological responses to visual, auditory, and combined stimulation

**DOI:** 10.3389/fnhum.2023.1286621

**Published:** 2024-01-08

**Authors:** Edoardo Maria Polo, Andrea Farabbi, Maximiliano Mollura, Luca Mainardi, Riccardo Barbieri

**Affiliations:** SpinLabs, Dipartimento di Elettronica, Informazione e Bioingegneria, Politecnico di Milano, Milan, Italy

**Keywords:** biomedical signal processing, emotions, International Affective Digital Sounds (IADS), International Affective Pictures System (IAPS), physiological responses, machine learning

## Abstract

Emotions significantly shape decision-making, and targeted emotional elicitations represent an important factor in neuromarketing, where they impact advertising effectiveness by capturing potential customers' attention intricately associated with emotional triggers. Analyzing biometric parameters after stimulus exposure may help in understanding emotional states. This study investigates autonomic and central nervous system responses to emotional stimuli, including images, auditory cues, and their combination while recording physiological signals, namely the electrocardiogram, blood volume pulse, galvanic skin response, pupillometry, respiration, and the electroencephalogram. The primary goal of the proposed analysis is to compare emotional stimulation methods and to identify the most effective approach for distinct physiological patterns. A novel feature selection technique is applied to further optimize the separation of four emotional states. Basic machine learning approaches are used in order to discern emotions as elicited by different kinds of stimulation. Electroencephalographic signals, Galvanic skin response and cardio-respiratory coupling-derived features provided the most significant features in distinguishing the four emotional states. Further findings highlight how auditory stimuli play a crucial role in creating distinct physiological patterns that enhance classification within a four-class problem. When combining all three types of stimulation, a validation accuracy of 49% was achieved. The sound-only and the image-only phases resulted in 52% and 44% accuracy respectively, whereas the combined stimulation of images and sounds led to 51% accuracy. Isolated visual stimuli yield less distinct patterns, necessitating more signals for relatively inferior performance compared to other types of stimuli. This surprising significance arises from limited auditory exploration in emotional recognition literature, particularly contrasted with the pleathora of studies performed using visual stimulation. In marketing, auditory components might hold a more relevant potential to significantly influence consumer choices.

## 1 Introduction

In decision making processes, a crucial role is played by the emotions experienced by each individual (Yadava et al., 2017). Within the field of neuromarketing, this cognitive process is particularly interesting as it can reveal which advertising stimuli can capture the attention of potential customers. Vlăsceanu (2014) confirms how this phenomenon is closely related to several aspects, including emotions.

To better understand the emotional characteristics of a subject, researchers study their biometric responses following specific stimuli. Particularly, a series of responses arise from the autonomous nervous system (ANS) and the central nervous system (CNS). The former is expressed through several physiological signals: electrocardiogram (ECG), blood volume pulse (BVP), eye tracking, galvanic skin response (GSR), and respiration (RESP) (Cuesta et al., 2019; Gill and Singh, 2020; Polo et al., 2022).

On the other hand, the central nervous system CNS is extensively investigated using different techniques for direct and indirect measurement of brain activity. The study of brain mechanisms and engagement following advertising stimuli are of great interest in literature and marketing studies, aiming to offer targeted and effective advertisements (Vences et al., 2020). Among the techniques for measuring CNS activity, the electroencephalogram (EEG) has gained success due to its non-invasiveness, ease of acquisition, and excellent temporal resolution in indirectly measuring cortical ensemble neuronal activity. This signal has been extensively studied in literature, and it is reported that cortical neural responses change over time, frequency, and space following emotional stimulation (Aftanas et al., 2002; Bamidis et al., 2009; Kheirkhah et al., 2020). Furthermore, from the EEG signal, attention and engagement indices can be obtained, which are useful in discriminating different types of emotions and their impact on the subject (Farabbi et al., 2022).

Indeed, each individual is capable of experiencing and expressing different emotions at varying intensities, and this becomes of great interest when understanding their emotional processes (e.g., strong emotions may influence a user's decision to proceed with a purchase). To classify different emotions and their intensity levels, multiple models are reported in the literature, among which Russells's model has been highly successful due to its simplicity (Posner et al., 2005). In this model, different emotions are depicted on a Cartesian graph with axes representing the level of arousal (emotion intensity) and valence (i.e., low level associated with unpleasantness and high level with pleasantness). The intersection of the two axes allows identifying individual emotions (e.g., happiness, fear, sadness, relaxation).

As mentioned before, the investigation of different emotions occurs through experiments involving stimulation. The stimulation phase requires robust protocols to study reliable responses. Most emotion stimulation protocols use images as stimuli, primarily sourced from the International Affective Picture System (IAPS) (Lang et al., 2005). Since the emotions elicited by these images are obtained from the labeling of thousands of people over the years, this database provides a robust standard for emotion stimulation protocols. Conversely, very few studies use auditory stimuli from the International Affective Digital Sound System (IADS) (Bradley and Lang, 2007). The IADS is analogous to the IAPS database, containing standardized sounds with valence and arousal levels.

Although a soundtrack in video clips is part of the stimulus itself, it is not easy to determine which type of stimulation, such as audio, visual, or audio-visual, is more effective in eliciting emotions. To the best of our knowledge, hardly any study uses a combination of these two types of stimulation.

Furthermore, in most studies, physiological signals or CNS-related signals are analyzed individually, limiting multimodal interactions (Anttonen and Surakka, 2005; Koelstra et al., 2012). In this article, we present a multimodal study of signals coming from the ANS and CNS following stimulation through images, sounds, and their combination. The complementarity of the different types of acquired and analyzed signals can contribute to a better understanding of the human body's response mechanisms to specific emotions and which signals are most useful for discriminating different emotions. Additionally, the different types of stimulation will be compared to understand which generate a more relevant response in terms of both physiological response and subject engagement. This comparison will also help to better understand which type of stimulation allows for better discrimination of different emotions. In the context of neuromarketing, this could be crucial in developing targeted advertisements for customers.

## 2 Materials and methods

### 2.1 Experimental protocol

The participants consisted of 13 females and 9 males with an average age of 26.18 ± 1.47 years. The experiments took place at the SpinLab of Politecnico of Milano, following the subjects' signed informed consent, which was approved by the Politecnico di Milano Research Ethical Committee (Opinion no. 29/2021). Throughout the experiment, pheripheral and central physiological signals were recorded, including ECG, BVP, GSR, pupillary signal (PUPIL), RESP and EEG. The ECG, GSR and RESP signals were recorded using Procomp Infiniti device, with a fixed sampling frequency of 256 Hz for GSR and RESP, and 2,048 Hz for ECG and BVP. The PUPIL signal was acquired using the Tobii Pro X2 Compact eye-tracker, with a sampling frequency of 60 Hz. The EEG signals were acquired using the DSI 24 headset, which consisted of 21 dry electrodes placed at specific locations based on the international 10–20 system: Fp1, Fp2, Fz, F3, F4, F7, F8, Cz, C3, C4, T7/T3, T8/T4, Pz, P3, P4, P7/T5, P8/T6, O1, O2, A1, A2. The EEG headset recorded data at a sampling rate of 300 Hz, using a 16-bit analog-to-digital converter.

Two extensively validated and widely used visual and auditory databases were selected for analysis. One database consisted of images (i.e., IAPS), while the other comprised sounds (i.e., IADS).

The protocol under examination extends a previously validated protocol in the literature (Valenza et al., 2014; Nardelli et al., 2015), which, however, was used separately with IAPS and IADS on two distinct samples. Nevertheless, it has shown promising results in terms of classifying valence and arousal dimensions when compared to other studies in the literature.

The experimental protocol consisted of three distinct and randomized phases with 2 min of rest in between aimed at investigating the efficacy of different types of emotional stimulation in eliciting distinguishable physiological patterns.

1) The first phase involved the presentation of visually stimulating images sourced from the IAPS database. In this phase, participants were exposed solely to visual stimuli, allowing for the examination of the physiological responses evoked by visual cues.

2) The second phase involved auditory stimulation, utilizing sounds extracted from the IADS database. Participants were exposed exclusively to auditory stimuli during this phase, enabling the investigation of the specific physiological patterns associated with auditory stimulation.

3) Lastly, in the third phase, a combination of visual and auditory stimuli was employed to create a more complex and integrated stimulus. In this phase images are presented alongside corresponding sounds carefully selected to have semantic congruence. This phase involved the simultaneous presentation of both IAPS images and IADS sounds, aiming to elicit a more comprehensive and nuanced emotional response by integrating visual and auditory sensory modalities.

**Figure 1:**
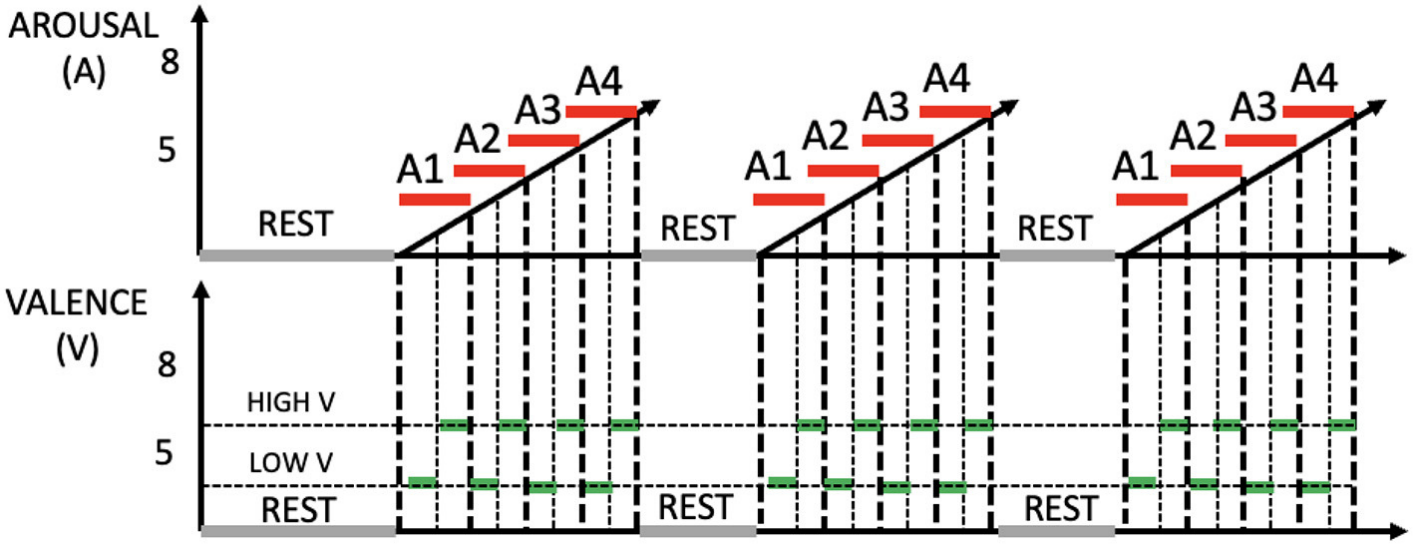
Outline of the experimental protocol, including three stimulation phases. **Top**: arousal (red). **Bottom**: valence (green).

By varying the type of emotional stimulation across these three phases, the study wants to determine the extent to which physiological patterns could be distinctively influenced by specific sensory inputs, thereby highlighting the interplay between visual and auditory modalities in emotional processing. The above-mentioned phases comprise four sessions, with each session lasting 90 seconds. Following an initial 5-min resting period where subjects view a grey screen, these sessions commence, progressively intensifying the levels of arousal (i.e., A1, A2, A3, and A4). Within each arousing session, there are six visual and/or acoustic stimuli, each with a duration of 15 seconds. These stimuli are partitioned into two halves, featuring low valence in the first half and high valence in the second half. The experiment's total duration spans 47 min. The underlying rationale for this design is to systematically elevate arousal levels while simultaneously maintaining a central neutral valence throughout each session. During the phase involving both visual and auditory stimulation, in addition to presenting images simultaneously with sound to create semantic matches, pairs of images and sounds were selected with comparable valence and arousal values. This choice was made to maintain the structure observed in the other phases, where arousal levels progressively increased, and low and high valences alternated within each arousal session. Arousal and valence levels are set according to IAPS and IADS scores as reported in Table 1. Figure 1 shows the experimental protocol. During the experiments, a designated experimenter remained present in the room at all times to address any potential issues with data acquisition and prevent subjects from making excessive movements or displacing sensors. Prior to the experiments, all participants underwent pure tone audiometry examinations using a clinical audiometer (Amplaid 177+, Amplifon with TDH49 headphones) to ensure that their hearing thresholds fell within the normal range (pure tone average thresholds at 0.5, 1, 2, and 4 kHz <20 dB HL). The auditory stimulation was delivered through earphones (UXD CT887), and participants with visual impairments were provided with the option to wear glasses. All participants were asked if they had any pre-existing medical conditions and/or mental disorders, and if so, they were excluded from the study. With regard to the visual aspects, and particularly concerning the pupillary signal, we followed the recommendations in the literature (Laeng and Endestad, 2012). To minimize external light interference, we decided to darken the laboratory windows and utilize artificial lighting during the recording. The screen brightness, on the other hand, remained constant for all subjects. As for the auditory component, the subjects were presented with three sounds before the commencement of the test, and they were instructed to select a volume level that they found comfortable.

**Table 1:** Valence and Arousal medians and ranges for all sessions (A1, A2, A3, and A4) of each phase (IAPS, IADS, and IAPS+IADS, which comprises Matched IAPS and Matched IADS).

**Session**	**Valence Rating**	**Arousal Rating**
	IAPS	
A1	4.96 (3.92–7.24)	4.68 (4.42–4.85)
A2	4.81 (2.52–7.62)	5.38 (5.08–5.48)
A3	5.10 (2.14–7.2)	6.55 (6.09–6.80)
A4	4.75 (1.45–7.57)	7.12 (6.90–7.35)
	IADS	
A1	5.16 (3.54–7.12)	4.77 (4.47–4.94)
A2	4.93 (2.46–7.78)	5.40 (5.05–5.87)
A3	4.88 (2.44–7.38)	6.35 (6–6.84)
A4	4.86 (1.68–7.67)	7.14 (7.03–7.88)
	matched IAPS	
A1	5.67 (3.65–7.13)	4.46 (4.13–4.75)
A2	4.44 (2.49–6.83)	5.43 (5.18–5.53)
A3	4.76 (2.16–6.83)	6.21 (6.06–6.79)
A4	4.19 (1.48–7.61)	7.18 (7.13–7.31)
	matched IADS	
A1	5.86 (4.52–7.05)	4.62 (4.38–4.87)
A2	4.82 (3.02–6.11)	5.50 (5.34–5.74)
A3	4.54 (2.06–6.77)	6.42 (6.07–6.64)
A4	4.54 (1.99–6.94)	7.35 (7.28–8.16)

### 2.2 Signal processing and feature extraction

The following section present the processing and extraction of features from the physiological signals recorded during the experiment, organized for each signal.

#### 2.2.1 The electroencephalogram

The EEG signals were imported and preprocessed using the EEGLAB toolbox in MATLAB. Initially, the data underwent filtering between 1 Hz and 45 Hz using a zero-phase finite impulse response filter. The Pz electrode recorded a faulty signal and was temporarily removed and later interpolated. Independent Component Analysis (ICA) with the Extended Infomax algorithm, as described in Lee et al. (1999) and Delorme et al. (2012), was applied to separate sub-Gaussian and super-Gaussian distributions using a natural gradient approach based on negentropy as a projection search index. The extracted components were categorized into seven classes (Brain, Eye, Muscle, Heart, Line Noise, Channel Noise, and Other) using the IClabel plugin (Pion-Tonachini et al., 2019) to assess their characteristics and origins. The accuracy of this categorization process was evaluated by analyzing the probability of component classification relative to brain or other sources. Artifacts were removed based on default threshold values, resulting in cleaned datasets for further analysis. The previously removed Pz electrode was interpolated using the spherical interpolation method. To minimize common noise, the Common-Average Referencing (CAR) method was implemented, subtracting the average potential of multiple electrodes from each individual electrode. The selection of the EEG signal processing pipeline was based on comparisons of different pipelines outlined in Cassani et al. (2022).

As the EEG signal is highly information-rich with numerous potential features, we concentrated on straightforward features that could be easily interpreted in order to evaluate any distinctions among the three types of stimulation. In this regard, the Power Spectral Density (PSD) was computed for the frontal and parietal regions across different frequency bands: δ (1–3 Hz), θ (4–7 Hz), α (8–12 Hz), and β (16–38 Hz). The PSD of each band has been normalized by the total power spectral density (i.e., 1-45 Hz). Numerous studies have unveiled the significance of these frequency bands within the EEG signal concerning emotions and the dimensions of valence and arousal (Sarno et al., 2016; Zhuang et al., 2017; Rahman et al., 2021). Analyzing the energy distribution within these frequency ranges thus provided a comprehensive understanding of patterns and trends related to different emotional states.

To assess the subjects' attention levels during the experiment, the ratio between the PSD in the β frequency band and the PSD in the θ frequency band was calculated for the frontal and parietal regions. These ratios, known as β/θ F and β/θ P, are recognized indicators of attention. It is believed that the ratios increase during attentive states, providing a more precise measure of attention throughout the trials (Cómez et al., 1998; Farabbi and Mainardi, 2022).

An engagement index (i.e., β/α) was also measured to assess whether the change in the type of stimulation affected not only attention but also the degree of involvement. The adoption of the β/α ratio as an engagement index is substantiated by its established effectiveness in a diverse array of empirical studies. This metric consistently proves its proficiency in gauging engagement across various contexts, including gaming tasks (McMahan et al., 2015), sustained attention tasks (Coelli et al., 2018), alarm-detection tasks (Dehais et al., 2018), and consumer behavior analysis (Kislov et al., 2022). Increased β power is associated with heightened brain activity during mental tasks, while increased α activity is related to lower levels of mental vigilance and alertness. The Power Spectral Densities were computed using the Welch method, a reliable and widely-used signal processing approach, to ensure accurate and reliable results in analyzing the EEG signals. In total, 6 features are computed, which include spectral power in different frequency bands as listed, an attention index, and an engagement index. These 6 features are calculated separately for both the frontal and parietal regions obtaining a total of 12 features for the EEG signals.

#### 2.2.2 The electrocardiogram

The ECG signal (sampled at a frequency of 2048 Hz) underwent initial processing, which included applying a fourth-order zero-phase low-pass Butterworth filter. Subsequently, a down-sampling operation was performed, reducing the signal to a rate of 250 Hz. The detection of R peaks on the ECG signal was achieved using the Pan-Tompkins algorithm (Sedghamiz, 2014). In order to obtain accurate measurements of heart rate variability (HRV), the Point process framework was employed. Since the temporal windows related to different emotional stimuli last 45 seconds within low- and high-valence arousal sessions, which is shorter than the conventional 5-minute windows typically used for extracting HRV measures (Sassi et al., 2015), the decision was made to utilize the Point process framework. This framework has proven to be robust in calculating HRV measures even in shorter temporal windows. Within the realm of statistics, point processes provide a probabilistic representation of the distribution of points within a specific space (Daley et al., 2003). These processes are commonly observed in diverse systems, including the temporal and spatial arrangement of neural spikes (Truccolo et al., 2005). The primary objective in the analysis of the ECG signal is to conceptualize heartbeats as a stochastic point process, facilitating the continuous estimation of the average inter-beat interval and related spectral indices. The behavior of heart cells, particularly during the initiation of an action potential, can be effectively m odeled as a Gaussian random walk with drift (Stanley et al., 2000). Consequently, the time interval between two successive heartbeats conforms to the Probability Density Function (PDF) of the Inverse Gaussian (IG), enabling real-time assessment of autonomic nervous system (ANS) activity (Barbieri et al., 2005; Chen et al., 2009, 2011). Through the point process framework a total of nine HRV features were computed: as the modelled RR series (μ*RR*) and the variability of the IG distribution (σ*2*), the RR power spectral density of the modelled RR series in very low (*RR VLF*) [<0.04 Hz], low (*RR LF*) [0.04–0.15 Hz] and high (*RR HF*) [0.15–0.5 Hz] frequency ranges, the sympatho-vagal balance index (*RR LF/HF*), the normalized power spectral density of the modelled RR series in low (*RR LFn*) and high (*RR HFn*) frequency ranges and the total power spectral density of the modelled RR series (RR TOT).

#### 2.2.3 Blood volume pulse

The BVP waveform illustrates blood volume fluctuations in a specific district (the finger) with each heartbeat. To analyze the BVP signal (sampled at 2,048 Hz), we applied a 4th-order low-pass Butterworth anti-aliasing filter with a cutoff frequency of 25 Hz. The signal was then downsampled to 250 Hz. Using the identified R-peaks in the ECG signal as a reference point, we extracted beat-to-beat systolic, diastolic, and onset fiducial points from the BVP signal. Systolic and diastolic values were determined by finding the maximum and minimum values between consecutive R-peaks, respectively. Onset values were pinpointed as inflection points between systolic and diastolic locations. We ensured precision through manual review and in-house software rectification. Analyzing the BVP and ECG signals, we derived two features: the Mean Volume Amplitude Index (VP), representing the mean amplitude difference between each systolic and its corresponding diastolic value, and the Mean Pulse Arrival Time (PAT), representing the mean temporal difference between each onset value on the BVP signal and its corresponding R-peak on the ECG signal. To compute PAT, we used onsets in relation to diastoles or systoles, as these points are more reliable and less susceptible to uncertainty in the signal's most tumultuous segments.

#### 2.2.4 Galvanic skin response

The GSR, also known as Electrodermal Activity, is a measure of skin electrical conductivity, reflecting two components: the Tonic Component representing the baseline skin conductance influenced by sympathetic nervous system activation, and the Phasic Component representing rapid fluctuations in electrodermal activity.

The GSR signal, sampled at a rate of 256 Hz, underwent a 4th-order low-pass Butterworth filter with a cutoff frequency of 2 Hz. Subsequently, it was downsampled to 5 Hz. To extract the Phasic Component, a median filter was applied, where each sample was replaced with the median value of the neighboring samples within a 4-second window centered around the current sample (Bakker et al., 2011). Subtracting this median signal from the filtered signal resulted in the Phasic Component. We chose to employ this method for computing the phasic component due to its straightforward yet effective capability in isolating rapid variations in the signal (Benedek and Kaernbach, 2010; Greco et al., 2016). Additionally, the signal was collected under conditions in which the subjects were seated and stationary, necessitating less extensive processing. The selection of this method was based on its simplicity and intuitive nature.

GSR peaks, corresponding to eccrine gland spikes, were identified by detecting local maxima in the filtered signal occurring between the onset (amplitude >0.01 μS) and offset (amplitude <0.0 μS) of the Phasic Component (Benedek and Kaernbach, 2010; Braithwaite et al., 2013). The analysis of the GSR signal resulted in the computation of seventeen features, encompassing averages and derivatives of the processed signal (Picard et al., 2001; Lisetti and Nasoz, 2004; Fleureau et al., 2012), as well as characteristics related to the number of GSR peaks and their relative timing (Kim and André, 2008; Frantzidis et al., 2010).

#### 2.2.5 Respiration

The respiration signal underwent a zero-phase digital low-pass filtering using the Parks-McClellan algorithm (Rabiner and Gold, 1975), with a cutoff frequency of 1 Hz, to isolate the desired frequency components. Following filtering, a thresholding technique identified maximum and minimum values in the signal. Two key features were extracted from the respiration signal: Respiratory Frequency (fRESP) and Mean Breath Amplitude (ampRESP). Mean Breath Amplitude was calculated as the difference between maximum and minimum values within each breath cycle. Additionally, a bivariate autoregressive point process model, akin to the univariate approach, was employed to estimate autonomic regulation of the heartbeat due to respiration-induced changes (Chen et al., 2009). This model separated the self-regulatory process from the effects of Respiratory Sinus Arrhythmia (RSA) on the ANS's feedback branch. The modeling allowed for time-frequency representations of the RR and RESP series, along with the corresponding cross-spectrum. It further facilitated the calculation of directional gains from respiration to heartbeat (RSA) and vice versa (Feedforward gains), providing high-resolution time-varying estimations of the average RR-interval. Furthermore, coherence in time and frequency was computed between RR and RESP. In total, eight features were computed from the RESP signal and its interaction with the ECG signal.

#### 2.2.6 Pupillometry

Despite the filtering options available in Tobii Pro Lab software, the raw data (sampled at 60 Hz) with blink compensation were chosen for further processing to preserve valuable information and apply a custom cleaning technique. Data points with diameters less than 2 mm or greater than 8 mm were designated as “NaN” to indicate blinks, as diameters outside this range are considered non-physiological. Artefacts due to acquisition errors were also removed: sudden increases or decreases of more than 0.375 mm within a 20 ms interval were identified and eliminated (Pong and Fuchs, 2000; Partala and Surakka, 2003). For blink compensation, sample replacement or interpolation was conducted for detected blink instances. If a blink was detected in one eye, the sample value for the other eye was substituted. If both eyes showed blinks, a cubic spline interpolation was used to estimate the missing data. This approach retained crucial information from the raw data. Next, a 4th-order zero-phase low-pass Butterworth anti-aliasing filter with a cutoff frequency of 5 Hz was applied to eliminate high-frequency noise. The filter was set to retain relevant signal information. Subsequently, the signal was downsampled to 10 Hz, an appropriate rate for pupillometric data analysis.

Finally, spectral analysis was performed using Welch's periodogram on the detrended signal. A 1.875-second Hamming window with a 50% overlap was used for the analysis. A total of nine features were computed, including the average pupil diameter and frequency features linked to the content of ANS.

### 2.3 Feature selection

To achieve simple and interpretable models for emotion classification, we adopted the Square Method (SM), which has a preliminary version described in Polo et al. (2021). The primary objective of this method is to identify the most significant features in the dataset that contribute to the optimal separation of the different classes within the 2D plane of features.

The process begins with the initial dataset comprising features' values and their corresponding labels. For each pair of feature values, 2D boxes are created, where the center of each box represents the average values of the features for different classes (i.e., emotions) under consideration. The sides of these boxes are determined by the 95% confidence intervals for the average estimation. The degree of overlap between boxes is computed by calculating the percentage of intersection area relative to the total area of each box. This process is performed for every pair of boxes, and then the average percentage is used as a metric to assess the importance of the feature pair. The lower the average intersection considering all intersections between boxes related to the two features, the higher the importance of this feature pair.

Below, we provide a detailed description of the algorithm for selecting the most important features in the classification process.

- For each class considered, 2D boxplots are created for all possible pairs of features.
- The algorithm computes the area of intersection between each pair of boxes and calculates the ratio between the area of intersection and the area of each rectangle. The average of all the ratios is then calculated and saved as A.
- For each feature pair, the algorithm checks if A exceeds a predefined threshold (e.g., 10%, 20%, and 30%). If A exceeds the threshold, the corresponding feature pair is discarded.
- Each feature is given a weight (W) based on the number of times it appears in the feature pairs not been discarded.
- The algorithm calculates the correlation between each feature pair, and if the correlation is greater than 80%, the feature with the lower W is discarded. If two features have the same W, one of them is randomly discarded.
- The remaining features are then sorted based on their W, indicating their importance. The higher the W, the more important the feature.
- The algorithm calculates the average of these W and considers only the features with a W greater than the average.

Through the examination of these boxplots, we can identify the features that frequently appear in pairs with minimal or no overlap between the boxes associated with different classes. Such features are deemed critical for emotion classification and are selected to serve as the foundation for the subsequent models.

**Figure 2:**
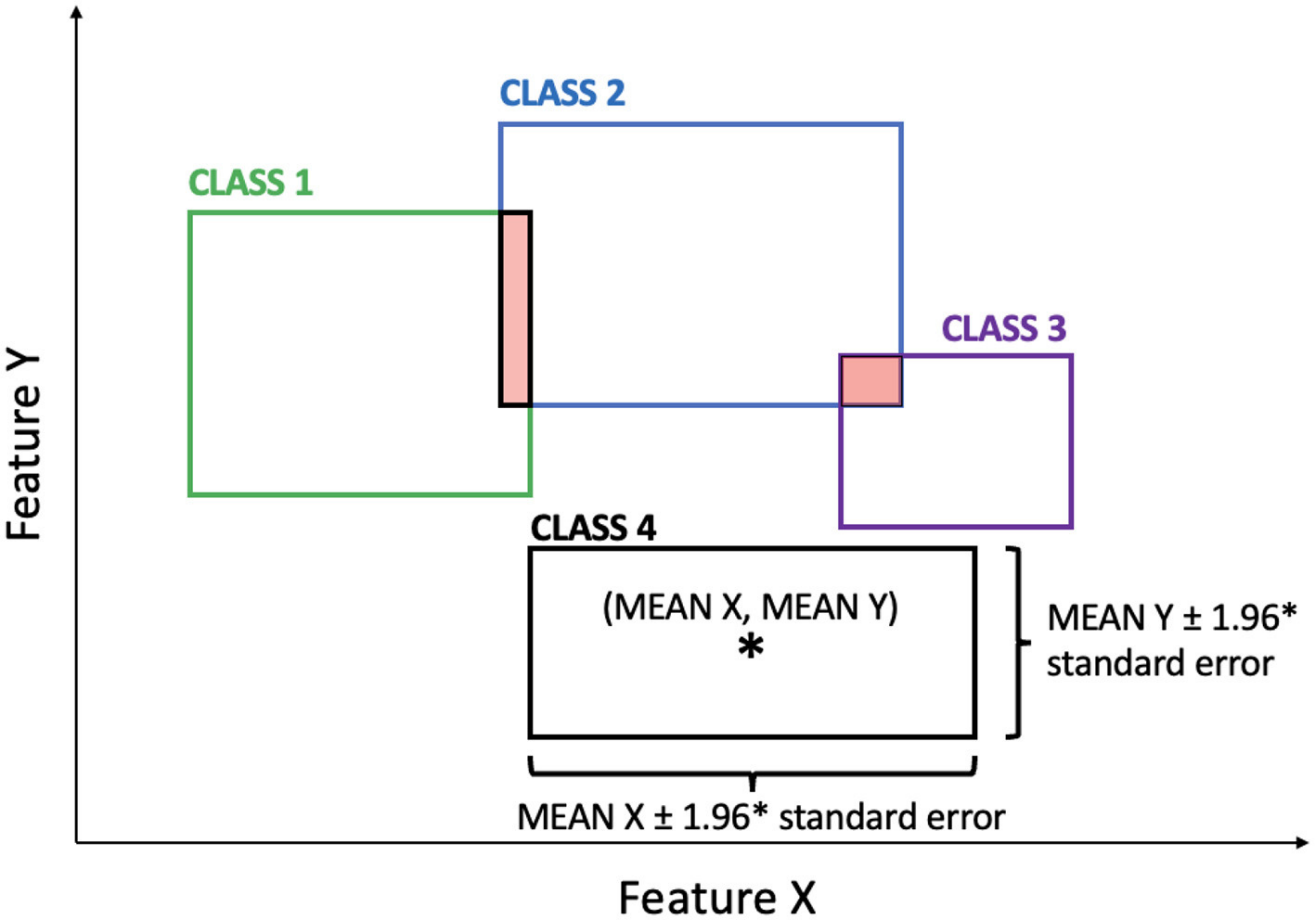
This example illustrates 2D boxes corresponding to a pair of features. Each box represents the values of the two features for a specific class. The overlapping regions between the boxes are highlighted in red, showing the intersections. ^*^Represents the center of each box, calculated as the average of the distributions of the two features within each class.

By employing the SM and analyzing the graphical representations, we can create streamlined and interpretable models that effectively capture the distinctive patterns in the feature space, enabling accurate emotion classification with a clear understanding of the underlying data structure. Figure 2 shows a possible example relative to a pair of features. As can be seen from the image, only two intersections are highlighted in red: one between class 1 and class 2, and the other between class 2 and class 3. In this specific case, only one class is completely separated from the others (i.e., class 4). In general, in this case, twelve intersections are calculated (i.e., the intersection of each box with all the others, 4 boxes * 3 possible intersections = 12 intersections). In the example, considering only the boxes related to class 2 and class 3, there are two intersection percentages, even though there is only one intersection. This is because each intersection is weighted by the total area of each box. Therefore, in this case, class 3 will have a higher percentage than class 2, as the area of class 3 is smaller.

### 2.4 Classification

A key goal of this investigation was to evaluate the generalization capabilities of machine learning models in the context of different types of stimulation and within each specific stimulation. Instead of prioritizing high-performance models, we opted for the utilization of simple machine learning approaches in conjunction with the transparent and easy-to-interpret feature selection method, denoted as SM. Our primary focus lay in gauging the effectiveness of these models in discerning emotions based on the type of stimulation applied. Furthermore, we sought to discern which types of stimulation led to a more distinct separation of emotions from physiological signals, and which types were less effective, giving rise to more ambiguous physiological experiences. To streamline the classification task, we excluded the two middle phases of increasing arousal (A2 and A3) for each type of stimulation. By concentrating on the extreme arousal states represented by phases A1 (very low arousal) and A4 (very high arousal), we aimed to facilitate the differentiation of emotions.

In total, the dataset encompassed 264 observations, with 12 samples per subject, representing all four quadrants of the Russell's cirumplex model of affect [i.e., first quadrant: A4-HV (Happiness/Amusement), second quadrant: A1-HV (Relaxation/Pleasure), third quadrant: A1-LV (Sadness/Depression), and fourth quadrant: A4-LV (Fear/Anger)], taking into account all three phases of stimulation (i.e., 22 subjects * 3 stimulation phases * 4 emotional quadrants).

For classification purposes, we abstained from applying dimension reduction methods to safeguard the integrity of the extracted signal features, ensuring that pertinent information was retained for the models. This approach allowed us to gain valuable insights into the discriminative power of simple machine learning models and feature selection techniques in discerning emotions under varying types of stimulation. As a result, our study aimed to provide a clearer understanding of the physiological responses to different emotional stimuli.

The dataset was divided into a training set (70%) and a test set (30%), with 7 randomly selected subjects excluded from the latter to avoid any data leakage. Subsequently, the customized SM feature selection method was applied to the training data to identify the most important features while minimizing correlation among them. The selection was performed using specific overlap thresholds for the 2D boxes, ranging from 100% (i.e., all uncorrelated features considered) to the minimum percentage required for at least one feature to be retained by the feature selection algorithm. This process aimed to achieve optimal feature subsets for distinguishing the four emotional states. Next, a 10-fold cross-validation, stratified by subjects, was conducted on the training set, employing different machine learning models, such as k-nearest neighbors (KNN), decision trees (DT), logistic regression (LR), support vector machine (SVM), linear discriminant analysis (LDA), random forest (RF), and Adaboost (ADB). A grid search was utilized to find the best combination of hyperparameters for each model. Subsequently, the top-performing models, based on the best percentage of overlap from the feature selection and highest average accuracy during validation, were retrained on the entire training set and then evaluated on the test set. Due to the dataset's balanced nature, the average accuracy during validation and the test accuracy were computed as performance metrics to assess the models' effectiveness in emotion classification.

Moreover, to assess the effectiveness of different types of stimulation in the classification process, the feature selection method was applied to three distinct datasets: images only, sounds only, and combined stimuli, each containing 88 observations. As before, specific overlap percentages were used during the feature selection process. Due to the limited number of observations in each dataset, only a 10-fold cross-validation, stratified by subjects, was performed for each dataset. The models were optimized using a grid search to identify the best hyperparameters. Subsequently, the average accuracy during validation was computed to evaluate the performance of the models for each type of stimulation.

Additionally, regarding the performance evaluation of the different models considered in the study, we focused on the best overlap percentage determined by the SM, which corresponded to the one yielding the highest performance for each stimulation. Subsequently, the performance of all models was assessed across all stimulations using this specific optimal overlap percentage.

## 3 Results

### 3.1 Machine learning performance

**Figure 3:**
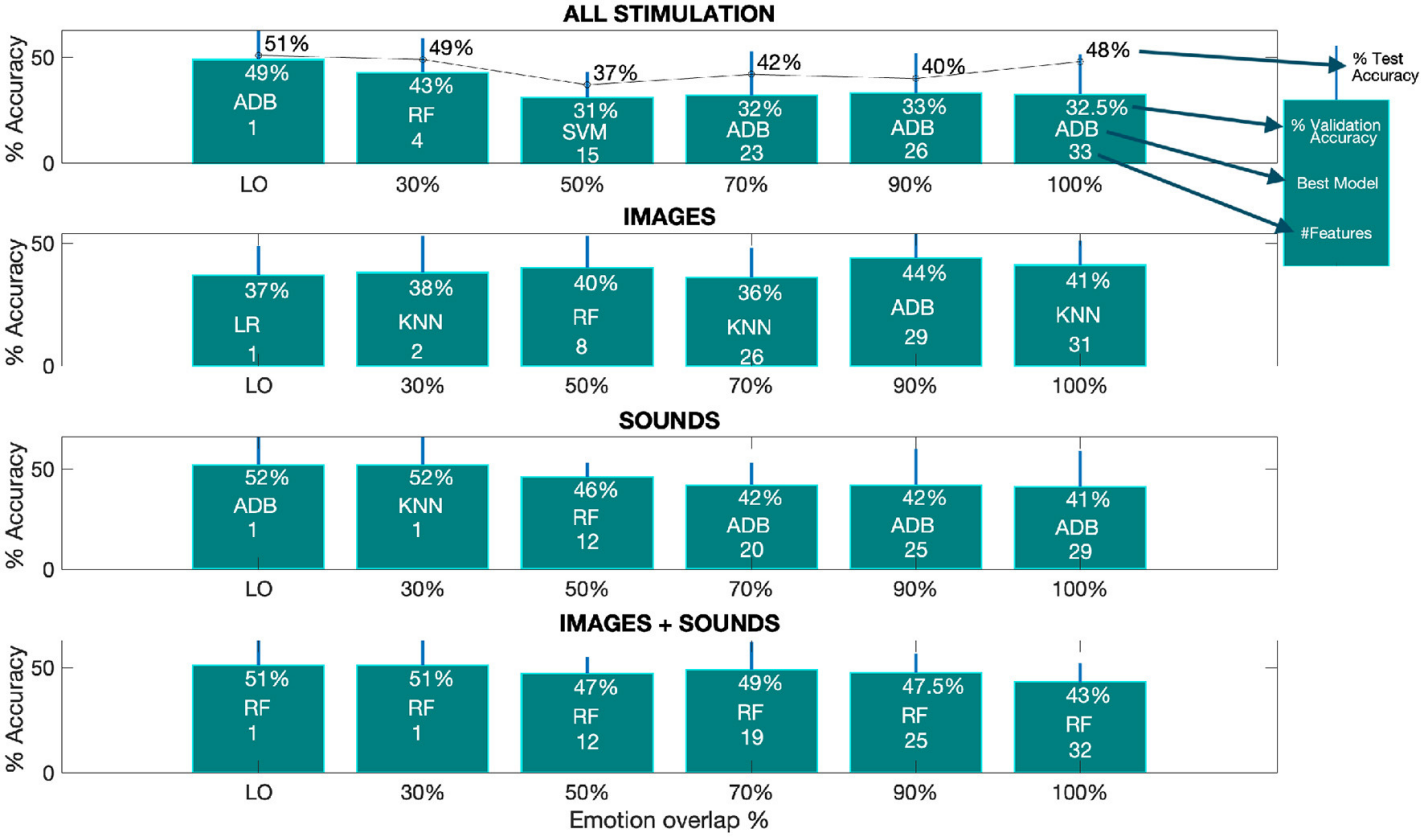
The optimal performance, as measured by accuracy, varies depending on the overlap percentage from the SM. The provided barplots displays the accuracy results achieved by the top-performing models at different overlap percentages for all classification tasks. These results are accompanied by the corresponding standard deviations, indicated by a line above each bar. Within each bar, you can find details about the percentage of validation accuracy, the specific model used, and the number of features selected from the SM for each overlap percentage. Additionally, in the first subplot, the percentage of test accuracy is also provided. The term “LO” denotes the lowest overlap percentage at which the SM still retains at least one selected feature. The corresponding lowest overlap percentages are as follows: 11% for all stimulation (test), 11% for all stimulation (validation), 24% for images (validation), 12% for sounds (validation), and 28% for images and sounds combined (validation).

Starting with the classification results for all types of stimulation together and separately, Figure 3 presents a summary of the best outcomes obtained across the different classifications performed. Specifically, the Figure displays the performance of the best model using the SM at each investigated overlap percentage, with the accuracy relative to the 4-class problem (i.e., random chance at 25%) depicted together with the model and the number of features used. As evident, as the overlap percentage decreases, the number of considered features also decreases.

From the Figure, we can deduce that the best performance for both test and validation data, when combining all types of stimulation, is achieved with the ADB model, with accuracies of 51% and 49% (first subplot - first barplot) at the lowest overlap percentage (LO), respectively.

From subplots 2 to 4, we observe the average validation accuracy for the three separated phases (i.e., images, sounds and image+sound). It is evident that, overall, higher accuracies are achieved during the sound and image+sound phases when compared to the image-only phase. The best average validation accuracy for the sound phase is 52% at the LO, while for the image+sound phase, it is 51% at the LO, both of which are higher than the 44% best accuracy obtained for images alone at 90% of overlap. Furthermore, apart from the image-only phase, the trend suggested by the average validation accuracy shows a decrease with an increase in the overlap percentage. This implies that as the overlap increases, more features are being incorporated into the classification process, leading to lower validation accuracy.

An important observation from the Figure is that the LO at which the SM obtains at least one feature, is similar for images (i.e., 24%) and images+sound (i.e., 28%), but notably lower for sound-only (i.e., 12%) and by joining all the stimulation together (i.e., 11%) as described in the caption of Figure 3.

### 3.2 Feature characterization

**Figure 4:**
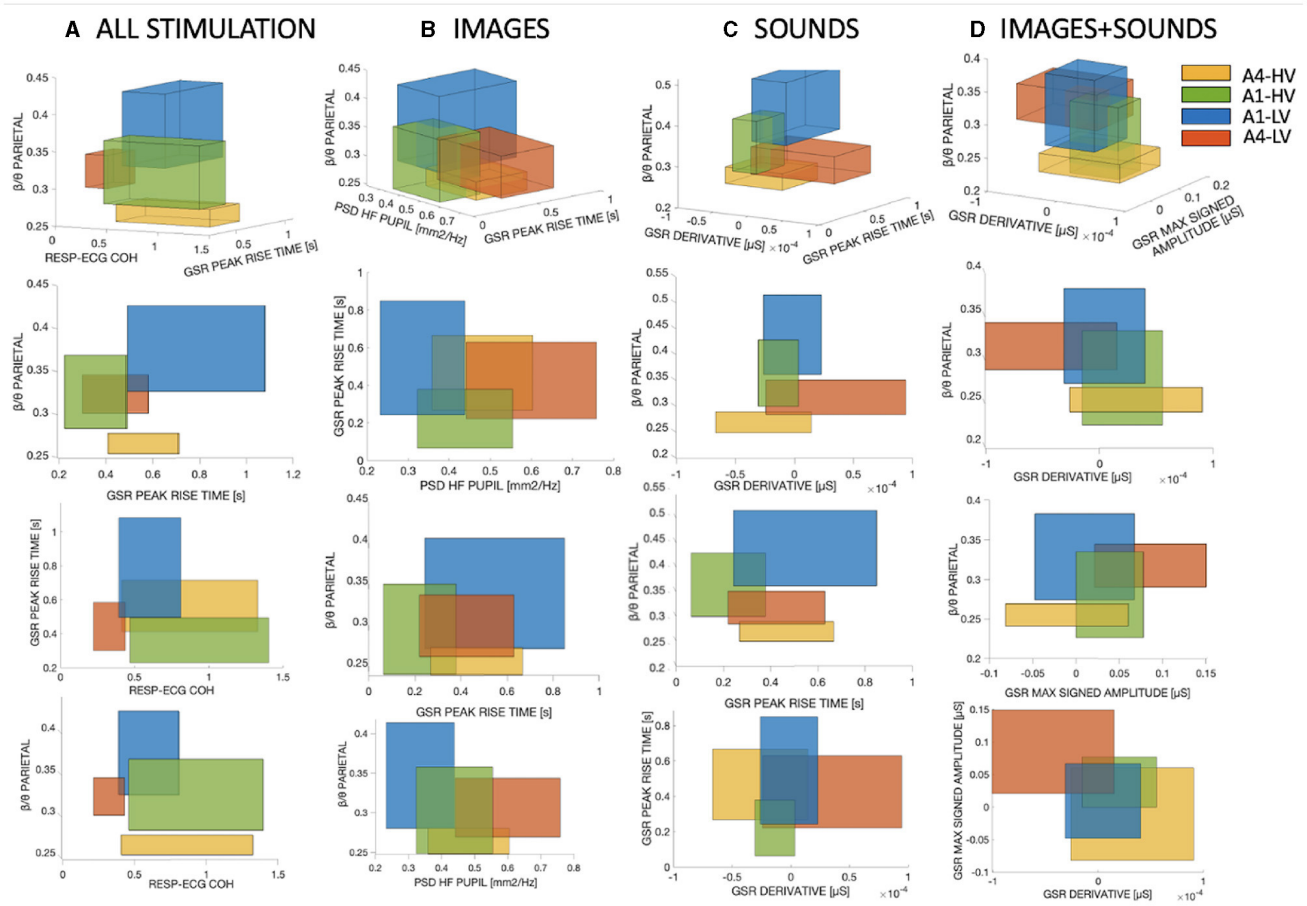
The 3D boxes were constructed using the three most important features obtained from the SM. **(A)** Corresponds to the combination of all three types of stimuli. **(B)** Represents the visual stimulation only. **(C)** Displays the auditory stimulation alone, and **(D)** Illustrates the combined visual and auditory stimulation.

To maximize the potential of the SM for visualizing the separation within the training observations across the four classes, 3D boxes were constructed using the three most important features identified by the SM. Additionally, projections between pairs of features were included. The process of creating the 3D boxes follows the same procedure as for the 2D boxes, with the addition of an extra feature. In Figure 4 four panels are presented: one integrating all stimuli together (1) and one for each stimulation method (2)-(4), each showcasing the 3D boxes.

From the Figure, it is evident that depending on the type of stimulation used, the intersections between different classes vary accordingly. Regarding Figure 4, the average overlap percentages were calculated for each class based on the three projections shown in the Figure. Table 2 presents the mean overlap percentage of the three projections of Figure 4 for each class, along with the overall mean considering all classes together. As observed, combining all stimuli results in lower overlap percentages. In general, we can observe that the sound phase exhibits the lowest overlap percentages compared to the three stimulations. Interestingly, it can be observed that for the sound and sound+image phases, the percentages are lower at higher arousal levels (i.e., A4), whereas the opposite trend is visible for the image-only phase.

**Table 2:** The average percentage of intersection overlap is computed for each emotional state in comparison to all other emotional states.

**Arousal/Valence**	**All**	**IAPS**	**IADS**	**IAPS+IADS**
**levels**	**stimulation**			
A1-LV	8%	13%	18%	32%
A1-HV	9%	27%	26%	40%
A4-LV	11%	26%	15%	19%
A4-HV	6%	30%	14%	20%
Average	8.5%	24%	18.25%	27.75%

This calculation involves utilizing the three most effective features for each type of stimulation with the SM.

In terms of feature importance, it is evident from Figure 4 that the parietal β/θ attention index consistently plays a crucial role in distinguishing the four classes, as it appears in all four panels and ranks among the top three best-performing features. This highlights the significant impact of the parietal β/θ attention index on the classification task, underscoring its importance as a strong predictor for separating the classes under study. Furthermore, referring to Figure 3, the β/θ feature is the only one that is not discarded by the SM even at low percentages. In fact, the LO in the Figure always corresponds to a single feature, which is the parietal β/θ. Figure 4 clearly shows that the emotion A1-LV (sadness/depression) is consistently associated with higher attention levels across all types of stimulation. There is a notable decreasing trend from A1-LV, A1-HV, A4-LV, to A4-HV, indicating a decrease in attention as arousal levels increase. This trend is particularly evident during the auditory-only stimulation, where the vertical overlap between the boxes is smaller, highlighting the significant importance of this attention index. In the combined stimulation, instead, A4-LV (fear/anger) surpasses A1-HV (relaxation/pleasure), indicating a shift in attention towards the valence dimension.

In addition to the attention index, another feature that has proven to be of significant importance for the separation of the four classes and subsequent classification is GSR PEAKS RISE TIME. This feature is computed over the found peaks as the average distance in seconds between the peak and the onset. Interestingly, this feature can be found among the three best-performing features in all types of stimulation, as well as when combining different types of stimulation, except for the image+sound phase. This particular feature appears to effectively distinguish the valence dimension at low arousal levels, as higher values consistently correspond to the emotion A1-LV, while lower values are associated with A1-HV.

Regarding the other features shown in Figure 4, which only appear in specific cases and are not recurrent, they are as follows:

RESP-ECG COH: This feature represents the coherence between the two time series of RESP and ECG. This feature appears to be more relevant when combining all types of stimulations (Figure 4A), and it specifically holds significant importance in distinguishing emotions along the valence dimension. Higher values are observed for emotions A1-HV and A4-HV compared to A1-LV and A4-LV.

PSD HF PUPIL: This feature is the Power Spectral Density of the Pupil Diameter in the high-frequency range (0.15–0.45 Hz). This feature appears to be relevant during the visual-only phase (Figure 4B), particularly in distinguishing the emotion A4-LV from the others. In general, this feature seems to be more closely related to the arousal dimension, with higher values associated with higher arousal levels.

GSR DERIVATIVE: This feature represents the average of the first derivative of the bandpass-filtered GSR in the frequency range of 0.5 to 1 Hz. This feature is relevant during the auditory-only phase (Figure 4C), especially for the emotion A4-LV, which is clearly distinct from the others. It is interesting to observe that this feature discriminates well between the two high arousal emotions, while there is a considerable overlap concerning the low arousal emotions, which lie in between A4-HV and A4-LV.

GSR MAX SIGNED AMPLITUDE: This feature represents the maximum signed amplitude between two consecutive extremes of the bandpass-filtered GSR in the frequency range of 0.5 to 1 Hz. It indicates the maximum range with a sign between consecutive maxima and minima. This feature has been found to be significant during the combined audio-visual phase (Figure 4D). Similar to GSR DERIVATIVE, GSR MAX SIGNED AMPLITUDE exhibits the same trend, effectively distinguishing the emotion A4-LV from the others and generally providing a clear division between high arousal and less pronounced division among low arousal emotions.

### 3.3 Feature importance and emotion classification

**Figure 5:**
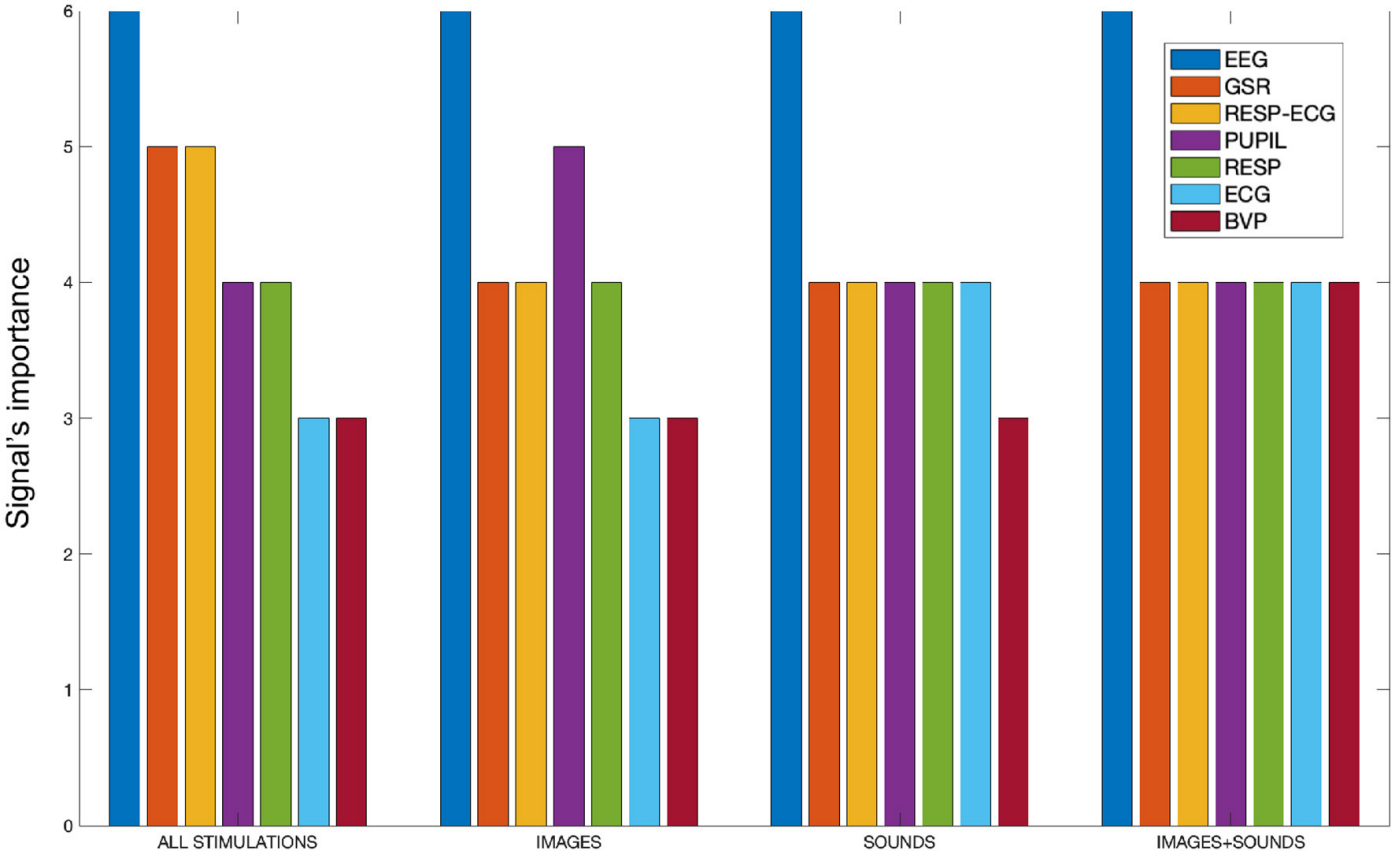
The bar plot illustrates the signal importance, quantified as the frequency of appearance of features related to each signal within the SM, categorized by stimulation type.

As discussed at the end of Section 2.4, to gain a comprehensive overview of the features and the signals used in the study, an analysis was conducted for each type of stimulation and considering all stimulations collectively. During this analysis, the frequency of features associated with each specific signal was calculated within the subsets of features retained by the SM for classification. This allowed us to determine the importance of each signal in the process of distinguishing the four emotions from each other. The frequencies were calculated on a scale from 0 to 6, where 6 represents the number of treated overlap percentages. The frequency measurement captured the presence of features linked to a specific signal that were not discarded by the SM and were consequently utilized in the classification process. As the frequency increased, it indicated that the features associated with a particular signal were not eliminated by the SM and were considered significant for classification purposes. This analysis allowed for the evaluation of the signal's importance in distinguishing the four emotions from each other. Essentially, a higher frequency suggested that the signal-related features were retained, reinforcing their role in effectively differentiating the emotional classes during the classification process. Figure 5 presents bar plots displaying the specific importance of each signal in the separation of emotions for each treated stimulation type. It was decided to include the “RESP-ECG” signal, as its related features pertain to cardio-respiratory coupling rather than just one of the two signals.

From Figure 5, it is evident that the EEG signal consistently maintains the highest frequency of appearance across all conducted stimulations, signifying its paramount importance compared to all other signals. Combining all stimulations together, the GSR signal and cardio-respiratory coupling seem crucial for separating the four emotions. Analyzing individual stimulations separately, we observe that the auditory and auditory+visual stimulations are quite similar, except for the BVP signal, which is considered one less time in the auditory-only stimulation. In the visual-only phase, besides the EEG signal, the pupillary signal also proves to be significant. Generally, the cardiovascular aspect appears to be the least performing among all. It is worth noting that there is an imbalance in the number of features calculated for each signal. For instance, the BVP signal, which appears to be the least significant from our analysis, also has the fewest features computed. However, it is important to clarify that we are not dealing with percentages. A signal is counted in our tally if at least one of its features remains within the feature set retained by the SM algorithm. Therefore, the importance of a signal is not influenced by the number of features considered; it is determined solely by whether at least one feature of that signal remains within the feature set retained by the algorithm.

### 3.4 Model performance and optimal feature selection

**Figure 6:**
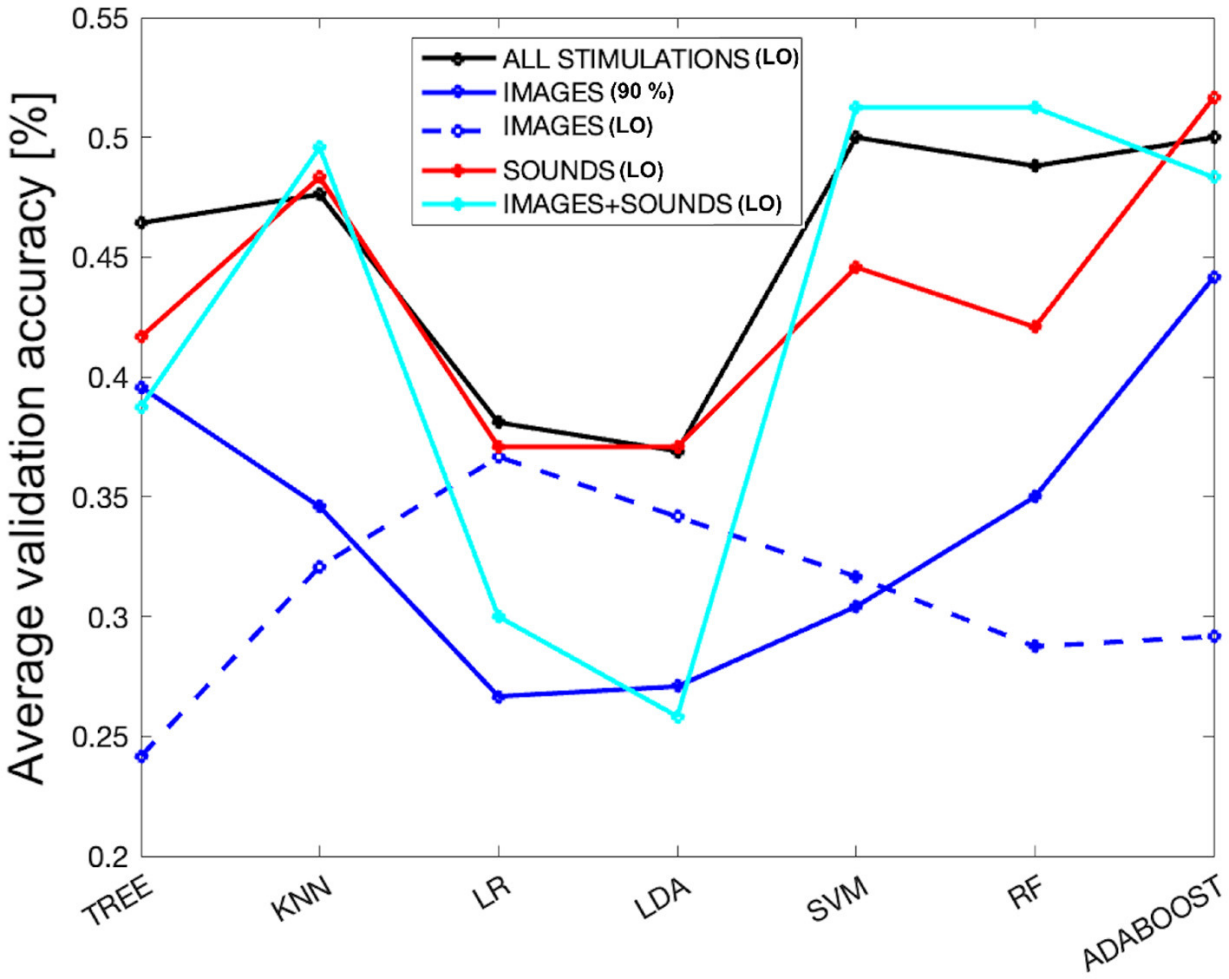
The average validation accuracy computed for all models and stimulations, using the features obtained from the best overlap percentages from SM. The dashed blue line represents the performance of the models using only the attention index derived from the EEG signal as a subset of features for the images-only phase.

As a final analysis, we evaluated the performance of the models by selecting the subset of features (derived from different overlap percentages) that resulted in the highest average validation accuracy across all considered stimulations. Referring to Figure 3, the best performance was achieved with an 11% overlap (i.e., 49% accuracy and one feature–first subplot) when combining all stimulations. For the image-only phase, the 90% overlap (i.e., 44% accuracy and 29 features–second subplot) performed the best. In the case of auditory stimulation, the 12% overlap (i.e., 52% accuracy and one feature–third subplot) yielded the highest accuracy, and for the combined audio-visual stimulation, the 28% overlap (i.e., 51% accuracy and one feature–last subplot) was the most effective. Figure 6 displays the average validation accuracy for all models and stimulations, utilizing the features resulting from the best overlap percentages.

In the Figure 6, it can be observed that LR and LDA are the worst-performing models across all types of stimulation, while KNN, SVM, RF, and ADB achieve better performances, with the tree-based models falling in between. KNN appears to be very stable, maintaining consistent performance across all types of stimulation except for images, where it notably drops. Overall, the performance for the images-only phase is the lowest among all the considered models. On the other hand, the performance in the sounds-only phase is the highest, although it shows quite comparable results to the sounds + images phase and the combination of all stimulations. However, there is a drastic drop in performance for the LR and LDA models during the images + sounds phase. Interestingly, the trend of different models for the sounds-only phase closely resembles that when considering all types of stimulation together. For completeness, as all types of stimulation, except for the images-only phase, achieved better performance using only one feature (i.e., the attention index derived from the EEG signal), the dashed blue line represents the performance derived from using only the attention index for the images-only phase. It can be observed that the images-only phase is the only one characterized by the lowest performance, even when using the same set of features for classification (resulting in a performance lower than the random chance, which is 25% for tree-based models). Interestingly, this phase shows an opposite trend compared to the best-performing model. In fact, LR and LDA models perform better in this specific case.

## 4 Discussions

The main goal of this study is to understand which type of stimulation is most effective in distinguishing between four different emotional states: viewing images only, listening to sounds only, or the combined action of both.

We harnessed the extensively validated IAPS and IADS datasets, both individually and concurrently, while overseeing a diverse array of physiological signals. Our study's distinctiveness stems from the novel application of these well-recognized datasets in investigating a variety of emotional stimulation methods. In contrast to the prevailing trend of focusing on visual stimuli in research, the incorporation of auditory elements remains an infrequent occurrence. Furthermore, the comparative underutilization of the IADS dataset when compared to the IAPS dataset serves as a unique characteristic of our study. Using both modalities with the same group of subjects is also uncommon. Furthermore, our research meticulously tracked six distinct physiological signals, adding depth to our analysis. The introduction of seven machine learning models introduces a new layer of complexity to our study, enabling us to evaluate their performance and effectiveness in emotion separation comprehensively. Our results originally portray a comparative demonstration of the significance of sound presence in creating more distinct and recognizable physiological patterns, leading to better and more separated features in the classification process.

### 4.1 Machine learning performance

From Figure 3, it is evident that the performance of the sound-only phase is the highest among all, though comparable to the combined audio-visual phase. The image-only phase, on the other hand, obtains significantly lower performance, with a maximum average validation accuracy of 44% compared to 52% for the sound phase. Furthermore, the Figure shows how, for the image-only phase, performance decreases as the number of features used for classification decreases, indicating that a greater number of features and, consequently, signals and information are required to achieve better performance. From a physiological perspective, this indicates that, following the protocol of this study, the vision of images alone creates less evident and distinct physiological patterns, necessitating the integration of many more signals and information to achieve better performance, which is still inferior to other types of stimulation.

Interestingly, for models relative to all types of stimulation combined, the best performance is achieved using only the attention index from the EEG signal, which has proven essential for classification, with a single feature. A comparable albeit diminished test performance is attained through the utilization of the maximum number of features (Figure 3–first subplot). In the first case, it is evident that sound and sound+image phases are more relevant in the classification, as they are well described by the attention index. However, increasing the number of features significantly raises the test performance due to the image phase, which requires more information.

Overall, it appears that the ADB model with only one feature (i.e., β/α P) performs the best when considering all stimuli together. This is evident in the test accuracy being very close to the validation accuracy (i.e., 51% and 49%, respectively). It implies that the test performance is minimally dependent on the specific test set used. On the other hand, under the condition of 100% overlap, a noticeable disparity between test accuracy (i.e., 48%) and validation accuracy (i.e., 32.5%) becomes apparent. This implies that the elevated test accuracy is predominantly influenced by the particular test set chosen. Nonetheless, when we assess all the scrutinized overlap scenarios, the ADB model consistently emerges as the frontrunner in 4 out of 6 instances. This finding underscores the increasing importance of this model, signifying its robust descriptive capability within the given context.

### 4.2 Feature characterization

Using SM as the algorithm for feature selection and importance, it was possible to find the features that best separate the four emotional classes. As mentioned earlier, the attention index in the parietal area was undoubtedly the most performing feature. It was the most important feature for all types of stimulation and proved crucial in separating emotions effectively for all types of stimulation. Physiologically (Figure 4, it was observed that the emotion A1-LV, related to sadness and/or depression, is associated with higher attention. This is in line with literature where negative stimuli tend to attract attention, presumably facilitating rapid threat detection (Iijima et al., 2018).

Of note, there is a decreasing trend for the attention index from low arousal (A1) to high arousal (A4), especially during the sound-only phase, where the separation concerning the attention index is distinct, except for the emotion A1-HV (relaxation/pleasure), as the others are almost completely separated. This observation suggests that emotions with lower arousal tend to elicit greater attention compared to more arousing emotions, except during the combined audio-visual phase. It is interesting to note that during the sound + image phase, the emotion of anger/fear (A4-LV) shows relevant higher attention compared to A1-HV, which was not the case for the sound-only and images-only phases. This evidence implies an increased focus on negative emotions compared to less arousing emotions during the audio-visual stimulation, indicating that the combination of sound and images tends to create more negative emotions concerning low-valence stimuli, raising the attention threshold.

Other features related to other physiological signals proved relevant for the separation of emotional states. Particularly, the GSR signal, with greater feature amplitudes usually associated with higher arousal (Scanlon and Sanders, 2018), showed clear differences, especially for A4-LV (Figures 4C, D), with considerably higher values compared to all other emotions. On the other hand, this behavior was not evident for the emotion A4-HV (happiness/amusement), which remained more intersected with low arousal states, obtaining even lower values. The AVG RISE TIME feature appeared to be more associated with valence, obtaining higher values for A1-LV and lower values for A1-HV (Figures 4A–C), suggesting that the rise time of eccrine gland spikes is generally longer for lower valence emotions.

Regarding cardio-respiratory coupling, coherence between the two series RR and RESP was also quite relevant, especially when all types of stimulation were combined. It is clear that coherence between the two series is higher for high valence and lower for low valence (Figure 4A). Thus, linear coupling between the two series is stronger during high valence emotional states, where emotions are positive and more relaxed.

The pupillary signal appeared relevant mainly during the image-only phase (Figure 4B), where spectral power density at higher frequencies seemed more linked to arousal, with higher values for A4-LV and A4-HV.

In general, from Figure 4 and Table 2, it is evident that the intersections between the different rectangles resulting from SM, using the three most relevant features for each stimulation, are smaller in the sound-only phase, which also shows the highest classification performance compared to the three phases of images, sounds, and combined audio-visual stimuli. The intersection is even smaller when considering all stimulations, primarily due to the fact that the number of observations used for calculating the boxes is three times higher than for individual stimulation.

In the realm of auditory engagement, emotional sounds undergo faster and more direct processing when compared to images, which necessitate intricate visual processing. This accelerated auditory system engagement could result in a more efficient stimulus for enhancing attentiveness (Jayaswal, 2016). Moreover, participants may perceive sounds as more remarkable and unexpected events since they exert limited control over the perception of auditory stimuli (Ivonin et al., 2013). Taking into account the insights gathered from multiple test participants, it was noted that when exposed exclusively to sounds devoid of any accompanying visual context, individuals had to focus their attention and rely on their past memories and experiences to comprehend the meaning of the sounds. Consequently, this led to the elicitation of deeper emotional responses.

### 4.3 Signals importance for emotions classification

As for signal importance, as shown in Figure 5, the EEG signal was the most important for each type of classification performed. Its importance was mainly attributed to the attention index, which was never discarded by SM and was always the top-ranked feature in importance, and using only this feature resulted in the best performance in the sound-only, sound + image, and all stimulation together. It is widely known that EEG signals have been a common focus of such developments compared to other physiological signals. Indeed, it was expected that it would reveal more pronounced differences in emotion recognition. This expectation arises from the fact that EEG directly informs us about brain function and has a shorter latency compared to other physiological signals, which naturally take longer to show noticeable changes. EEG facilitates real-time monitoring of brain activity and provides rich information with high temporal precision (Rahman et al., 2021). Moreover, the ability to target specific cortical regions using electrodes allows for extracting abundant data related to different cortical areas with distinct functions based on their location. This capability aids in exploring brain hemisphere-related asymmetries, contributing to a more comprehensive understanding of emotional processing (Niemic, 2004).

Despite this, other signals have proven important in separating the four emotional states, such as GSR and features derived from cardio-respiratory coupling while only during the image-only phase, the pupillary signal was relevant. Regarding the GSR signal, it is well-established that it is closely related to sympathetic responses and, consequently, arousal (Scanlon and Sanders, 2018; Wang et al., 2018). As observed in Figure 4, its power lies indeed in its ability to effectively distinguish between low and high arousal emotions. A similar observation can be made for features related to cardio-respiratory coupling, even though the literature supporting it is less extensive. The relevance of these features might be attributed to the widely accepted association of RSA with parasympathetic activity. An increase in RSA is generally considered an indicator of heightened parasympathetic activation, a relationship established in previous research studies (Grossman and Svebak, 1987; Frazier et al., 2004). This increased parasympathetic influence could have contributed to the effective differentiation of emotional states. As for the pupillary signal, it has exhibited greater significance during the phase involving solely images, where the visual sense was most stimulated.

Cardiovascular signals ECG and BVP were the least relevant for emotional state separation and subsequent classification. Despite using the point process framework, which allowed calculating real-time HRV indices even in short time windows (i.e., 45 seconds) and thus more robust features compared to traditional methods that require at least 5 min of recording, they failed to capture different activation patterns according to the different emotional states.

### 4.4 Model performance with best feature selection

Regarding the performance of machine learning models, it was observed that the image-only phase performed significantly worse. From Figure 6, it is clear that the blue line representing the image phase, for all models, is consistently lower than the other types of stimulation. In particular, the sound phase (red line) shows a very similar behavior to when all stimulations are together, indicating that the sound phase plays a more prominent role in classification. In general, sounds are characterized by less variability in performance among different models compared to the other two phases, indicating a more robust performance even with varying model families. Concerning the image-only phase, it can be concluded that it obtained the worst performance, even with an equal number of considered features. The dashed blue line, which represents the performance of the image phase using only the attention index, achieved very low performance, and the maximum performance obtained with LR is the minimum performance for the sound-only phase. This suggests that sounds played a crucial role in separating emotions and subsequent classification, demonstrating that they create more distinct physiological patterns concerning emotional states. This finding is particularly intriguing, given the relatively limited exploration of the auditory dimension in emotional recognition literature as compared to its visual counterpart (Gerdes et al., 2014).

Moreover, our results showed that within the domain of marketing, sounds may possess the potential to wield a more pronounced influence over consumer decisions, emphasizing their importance as an avenue for further investigation and application.

In relation to the models employed in this study, our selection was guided by the prevalent machine learning models extensively documented in the literature (Bota et al., 2019). Specifically, KNN and RF have shown remarkable performance in the realm of emotion classification via physiological signal analysis in numerous prior investigations (Kolodyazhniy et al., 2011; Rubin et al., 2016; Myroniv et al., 2017; Pinto et al., 2020). In our case as well, KNN and RF emerge as compelling options, as evidenced in Figure 3, where a majority of the models yield superior results, particularly when considering varying levels of overlap.

Furthermore, in Figure 6, we observe that when utilizing the feature set associated with the best overlap percentage, both KNN and RF consistently exhibit strong performance. Notably, KNN demonstrates minimal variability across different stimulation conditions, be it sound-only, image+sound, or all stimuli together. Conversely, RF displays more variability, with a more significant decline in performance during the sound-only phase but excelling in the image+sound classification, as indicated in the last subplot of Figure 3 where RF is always chosen as the best model varying the overlap percentage.

Nevertheless, it is crucial to highlight that ADB consistently outperforms its counterparts. ADB has recently gained substantial recognition within the academic literature for its role as an integrated algorithm that effectively constructs robust classifiers through the iterative aggregation of weak classifiers. Recent empirical investigations have underscored the remarkable efficacy of ADB in the context of multiclass emotion recognition tasks (Zhang et al., 2018; Chen et al., 2021). In our own comprehensive study, ADB consistently achieved the highest average performance, as depicted in Figure 3. Exceptionally, during the image+sound phase, RF showcases outstanding performance. Nevertheless, ADB outperforms in the image-only, sound-only, and combined stimulus phases. As delineated in Figure 6, ADB's performance remains comparable to RF also in the image+sound phase (i.e., 48% of validation accuracy with respect to 51%, respectively), further underscoring its reliability and effectiveness. Furthermore, even when confronted with varying degrees of overlap, ADB maintains its prominent presence among the selected models. Hence, based on the empirical evidence at hand, ADB unequivocally emerges as the optimal choice for the task of emotion classification.

## 5 Conclusions

In conclusion, this study provides novel, valuable insights into the role that different stimulation modalities have in eliciting emotional states at a physiological level. An original, comprehensive comparative protocol allows to analyze the interrelationships among three different stimulation modalities and four different emotional states through the performance of a wide range of machine learning models in discriminating between emotional states. The final outcome underscores the pivotal role of sound in creating distinct physiological patterns and its impact on classification accuracy. Notably, the EEG signal emerges as the most critical feature for classification, while other physiological signals such as GSR and cardio-respiratory coupling also prove relevant in delineating emotional states.

Regarding the machine learning models employed, the ADB model emerges as the preferred choice, consistently achieving the highest accuracy across various stimulation types. It attains peak performance in the image-only phase (i.e., 44%), sound-only phase (i.e., 52%), and the combined stimuli phase (i.e., 51% in the test set and 49% in the validation set). Even in the image+sound phase, where RF records the highest accuracy (i.e., 51%), ADB exhibits comparable performance (i.e., 48%), solidifying its position as the model of choice among the options considered.

Looking forward into the near future, more elaborate protocols could explore the incorporation of additional physiological signals, refine analytical techniques to enhance their effectiveness in the realm of emotional state recognition, and increase the sample size to bolster the robustness of results. Furthermore, investigating how responses vary across different stimuli at the neutral level, considering both valence and arousal, offers a promising avenue for exploration.

These findings hold potential implications in diverse domains, including emotional recognition and marketing strategies.

## Data availability statement

The unidentified aggregate version of the data supporting the conclusions of this article will be made available under request by the authors, without undue reservation.

## Ethics statement

The studies involving humans were approved by Comitato Etico del Politecnico di Milano. The studies were conducted in accordance with the local legislation and institutional requirements. The participants provided their written informed consent to participate in this study.

## Author contributions

EP: Formal analysis, Investigation, Methodology, Writing—original draft. AF: Investigation, Methodology, Writing—original draft. MM: Supervision, Writing—review & editing. LM: Supervision, Writing—review & editing. RB: Supervision, Writing — review & editing.
